# Oxidative stress indices in ASD children in Sub-Sahara Africa

**DOI:** 10.1186/s11689-021-09379-w

**Published:** 2021-10-19

**Authors:** Ishiaq Olayinka Omotosho, Adekunbi Olufunke Akinade, Ikeoluwa Abiola Lagunju, Momoh A. Yakubu

**Affiliations:** 1 Department of Chemical Pathology, Neurotoxicology Unit, University of Ibadan, Ibadan, Nigeria; 2 Department of Paediatrics, College of Medicine, University of Ibadan, Ibadan, Nigeria; 3 Department of Environmental & Interdisciplinary Sciences, Texas Southern University, New Science Building Suite, Houston, TX 303 USA

**Keywords:** Essential and toxic metals, Oxidative stress, Autism spectrum disorder, Imbalance in oxidant/antioxidant ratio

## Abstract

**Background:**

The pathogenesis of autism spectrum disorder (ASD) remains a medical challenge even in the developed world. Although genetics and epigenetic factors have been variously indicted as major causes of the disorder, development of oxidative stress especially in the formative years of children has equally gained prominence as an etiological basis of the disorder. Oxidative stress is characterized by the production of excessive amounts of free radicals, decreased levels of antioxidants with the attendant imbalance in oxidant/antioxidant ratio. This study was designed to determine the levels of essential metals [magnesium (Mg), zinc (Zn), and copper (Cu)] and toxic metal, lead (Pb), and generation of oxidative stress by their abnormal interaction.

**Method:**

Twenty-five children clinically diagnosed for ASD according to DSM-IV-TR and 25 neuro-typical (NT) children (controls), (aged 5.96 ± 1.40 years and 6.18 ± 2.59 years respectively) were recruited for this study. Essential and toxic metals were analyzed using induction-coupled plasma-mass spectrometry (ICP-MS); oxidative stress markers [malondialdehyde (MDA), total plasma peroxidase (TPP), and total antioxidant capacity (TAC)] were determined using appropriate biochemical methods. Oxidative stress index (OSI) was calculated.

**Results:**

The levels of TPP and TAC were significantly reduced while MDA was higher in ASD compared to NT. Although OSI was higher in ASD, the difference was not significant. Pb (lead) concentration was significantly increased while Mg, Zn, and Cu levels were reduced significantly in ASD compared to NT. A significant negative correlation between Mg and OSI (*r* = − 0.438; *p* = 0.029) was observed in NT.

**Conclusion:**

Reduction in Zn and Mg levels with a concurrent increase in Pb in children with ASD in this study may be the basis of inadequate TAC manifesting as increased MDA and reduced TPP levels. The attendant imbalance in oxidant/antioxidant ratio may result in abnormality in neuronal transduction leading to the abnormal cognitive and speech functions characteristic of ASD.

## Introduction

Autism spectrum disorder (ASD) is a neurodevelopmental disorder that is becoming increasingly prevalent. ASD is a heterogeneous group of neurodevelopmental disorders characterized by impairments in verbal and non-verbal expressive speech, deficits in social interaction and hyper-focused repetitive behaviors [1, 2]. The causes and pathophysiology of ASD are not fully understood. There is a general agreement that ASD could result from interaction between genetic and environmental factors with oxidative stress as a potential link [3]. Oxidative stress has recently been linked to the etiology of this disorder along with multiple genetic and environmental factors [4, 5]. Oxidative stress is characterized by the production of excessive amounts of free radicals, decreased levels of antioxidants, or both. Excessive production of free radicals or impaired antioxidant mechanism may cause oxidative stress which may induce several pathophysiological processes. Cellular antioxidant defense mechanism prevents the generation of free radicals and inactivates them after generation. Impaired antioxidant defense mechanism can result in cell membrane damage, alteration in membrane fluidity and permeability, and oxidative changes in proteins, lipids, and DNA [6]. Several environmental factors like heavy metals particularly Pb have been implicated in ASD. Understanding the levels of anti-oxidative/oxidative status in children with ASD may assist in clarifying the biochemical mechanisms underlying the pathophysiology of these disorders. That the pathophysiology of ASD involve oxidative stress resulting from exposure to heavy metals as environmental pro-oxidants has been reported [6]; however, there are limited or no information on this aspect of the disorder in sub-Sahara Africa. This write-up, therefore, examines the impact of exposure to some toxic and essential metals on levels of oxidative stress markers/indices in the development of ASD using black African children as a model.

### Subjects and method

A total number of 50 participants were recruited into this study. This comprised 25 children clinically diagnosed for ASD (5.25 ± 0.37 years) as cases by specialists in child neurology and child psychiatry after proper evaluations and diagnosis using Diagnostic and Statistical Manual of Mental Disorder (DMS-IV-TR) classification and 25 neurotypical (NT) children (6.18 ± 2.2.59 years) as controls. The control were recruited from friends, colleagues, and neighbors, unrelated to the case group. All participants recruited for this study received routine childhood vaccinations/immunization.

### Ethical approval

Ethical approval was obtained from the UCH/UI joint Ethical Committee (UI/EC/15/0087) and Oyo State Ministry of Health Ethical Board. Informed consent was obtained from each participant by proxy.

#### Inclusion criteria

Clinically diagnosed children with ASD that their parents gave informed consent were recruited for the study.

#### Exclusion criteria

Participants that were suffering from liver or kidney disease, anemia, or current treatment for iron deficiency, progressive neurological disorders, or epilepsy were excluded from the study. None of the participants was on psychotropic drug.

Blood sample was collected randomly from each participant from the ante-cubital vein. Blood was carefully dispensed to avoid hemolysis into lithium heparin bottles. Blood samples were centrifuged at 2000 r.p.m. at room temperature for 10 min using centeur 2-centrifuge (Fiston centrifuge, manufactured in England) to obtain plasma.

All samples were kept frozen at – 80 ^o^C and were later analyzed for metals and oxidative stress markers.

Essential and toxic metals were analyzed using ICP-MS. Level of malondialdehyde (MDA) was estimated using the method of Adam-Vizi and Seregi [7], Total plasma peroxidase (TPP) was estimated using Fox-2 reagent as described by Uma Devi et al. [8] with minor modifications by Harma et al. [9] and total antioxidant capacity (TAC)] was determined using ferric reducing antioxidant powder (FRAP) reagent as described by Benzie and Stram [10] while oxidative stress index (OSI) was calculated as the ratio of total peroxide to the total anti-oxidant capacity.

Statistical analysis was done using Statistical Package for Social Science (SPSS 20. 0.1 for windows; SPSS Inc., Chicago, IL, 2016).

#### Descriptive statistics

Mean standard error (± SEM) was used for parametric numerical data, frequency, and percentage for non-numerical data.

#### Analytical statistics

Student’s *t* test was used to assess the statistical significance of the difference between two study groups means.

ANOVA test was used to assess the statistical significance of the difference between more than two study groups means.

Chi-square test was used to examine the relationship between two qualitative variables. Fisher’s exact test was used to examine the relationship between two qualitative variables when the expected count is less than 5 in more than 20% of cells.

*P* value of 0.05 was considered significant.

## Results

### Table 1 is a summary of the biodata variables between the autism spectrum disorders and neuro-typical children

The results showed no significant differences in all the biodata variables except the birth weight that showed significantly increased birth weight for ASD compared to NT (*p* = 0). Although, there was a difference in birth weight of cases and controls, the two groups were comparable based on other biodata obtained through structured questionnaire.

**Table 1:** Comparison of Biodata and Biochemical Variables Between ASD and NT Groups Using Student T- Test

VARIABLES	ASD (*N* = 25)	NT (*N* = 25)	*t*-VALUE	*P*-VALUE
**Child’s Age (yrs)**	5.96 ± 1.45	6.18 ± 2.2.59	− 0.370	0.713
**Mother’s Age at Birth (yrs)**	26.68 ± 2.69	27.96 ± 2.70	−1.680	0.100
**Father’s Age at Birth (yrs)**	31.72 ± 2.98	32.32 ± 3.86	−0.615	0.541
**Number of Siblings**	1.72 ± 0.79	1.60 ± 0.91	0.497	0.622
**Child’s weight (kg)**	19.64 ± 3.99	19.00 ± 5.18	0.490	0.627
**Mg (mg/dl)**	2.53 ± 0.46	3.13 ± 0.43	−4.830	0.000*
**Zn (μg/dl)**	222.3 ± 63.8	438.5 ± 185.5	−5.511	0.000*
**Cu (μg/dl)**	4.32 ± 1.02	4.88 ± 0.94	−2.020	0.049*
**Zn/Cu**	55.31 ± 22.04	92.29 ± 44.57	−3.719	0.001*
**Pb (μg/dl)**	9.49 ± 4.04	5.43 ± 2.04	4.483	0.000*
**Total Plasma Peroxidase (TPP)**	105.9 ± 2.3	110.4 ± 7.9	−2.679	0.010*
**Total Antioxidant Capacity (TAC)**	280.2 ± 34.4	303.8 ± 33.1	−2.468	0.017*
**Malondialdyde (×10**^**−5**^**) (MDA)**	2.27 ± 0.23	1.42 ± 0.13	16.127	0.000*
**Oxidative Stress Index (OSI)**	0.38 ± 0.05	0.37 ± 0.05	1.083	0.284

*Significant at *p* < 0.05

Also, the summary of mean ± SEM of essential trace metals and toxic metal between ASD and NT showed significant differences in all the biochemical parameters. Mg, Zn and Cu were significantly reduced in ASD compared to NT (*p* < 0.000; *p* < 0.000 and *p* < 0.049) respectively. Zn/Cu ratio was also significantly reduced in ASD compared to NT (*p* < 0.001). Pb on the other hand was significantly higher in ASD than in NT (*p* < 0.000). Aside this, mean ± SEM levels of oxidative stress markers in ASD and NT showed that TPP and TAC were reduced (*p* < 0.010; *p* < 0.017) while MDA was higher in ASD compared to NT (*p* < 0.000). Although OSI was higher in ASD, the difference between the two groups was not significant (*p* > 0).

### Table 2: Correlation of levels of essential and toxic metals level with oxidative markers in NT

There were no significant correlations among the trace metals (including Pb) and oxidative markers in Neurotypical children. However, expressing concentration of Cu as a ratio of Zn showed that Mg positively correlated with Zn/Cu ratio (*r* = 0.589; *p* = 0.002). Cu had negative correlation with Zn/Cu ratio (*r* = -0.785; *p* = 0.000) while Zn positively correlated with the ratio (0.839; *p* = 0.000).

**Table 2:** Correlation of levels of essential and toxic metals level with oxidative markers in NT

		TPP	TAC	MDA	OSI	Zn/Cu
Mg	r	−0.182	0.058	0.006	−0.092	0.589
	P	0.384	0.784	0.979	0.663	0.002*
Zn	r	−0.128	0.147	0.260	−0.172	0.839
	p	0.542	0.483	0.209	0.411	0.000*
Cu	r	0.384	0.202	0.095	−0.151	− 0.785
	p	0.058	0.334	0.653	0.472	0.000*
Pb	r	−0.109	−0.105	−0.036	0.039	0.048
	p	0.602	0.619	0.866	0.853	0.821

*Significant at *P* < 0.05

### Table 3 is a summary of correlation analysis between trace metals (including Pb) and oxidative stress markers in ASD children

There was a significant negative correlation between Mg and OSI (*r* = -0.438; *p* = 0.029) and a significant positive correlation between Zn and Zn/Cu ratio (*r* = 0.907; *p* = 0.000).

**Table 3:** Correlation of toxic and essential metals levels with oxidative stress markers in ASD

		TPP	TAC	MDA	OSI	Zn/Cu
Mg	r	− 0.337	0.291	0.284	−0.438	0.378
	p	0.099	0.158	0.169	0.029*	0.063
Zn	r	0.055	0.158	0.192	−0.106	0.907
	p	0.793	0.452	0.357	0.613	0.000*
Cu	r	0.222	−0.077	− 0.133	0.170	− 0.302
	p	0.287	0.715	0.527	0.417	0.142
Pb	r	−0.195	−0.165	0.303	−0.004	0.238
	p	0.350	0.431	0.140	0.986	0.252

*Significant at *p* < 0.05

### Table 4: Correlation of Zn/Cu ratio with Oxidative indices in Children with ASD

No significant correlations were observed among markers of oxidative stress including with Zn/Cu ratio in children with ASD except between OSI and TAC (*r* = -0.983, *p* = 0.000) where a highly significant negative correlation was obtained.

**Table 4:** Correlation of Zn/Cu ratio with oxidative indices in children with ASD

Correlations in ASD						
		TPP	TAC	MDA	OSI	Zn/Cu
TPP	Pearson Correlation	1	−.083	.041	.225	−.180
	Sig. (2-tailed)		.692	.846	.279	.389
	N	25	25	25	25	25
TAC	Pearson Correlation	−.083	1	.360	**-.983**^**a**^	−.093
	Sig. (2-tailed)	.692		.077	**.000**	.659
	N	25	25	25	25	25
MDA	Pearson Correlation	.041	.360	1	−.369	−.039
	Sig. (2-tailed)	.846	.077		.069	.852
	N	25	25	25	25	25
OSI	Pearson Correlation	.225	**-.983**^**a**^	−.369	1	.065
	Sig. (2-tailed)	.279	**.000**	.069		.759
	N	25	25	25	25	25
Zn/Cu	Pearson Correlation	−.180	−.093	−.039	.065	1
	Sig. (2-tailed)	.389	.659	.852	.759	
	N	25	25	25	25	25

^a^Correlation is significant at the 0.01 level (2-tailed)

### Table 5: Correlation of Zn/Cu ratio with Oxidative indices in NT Children

The results of correlation analysis of markers of oxidative stress and Zn/Cu ratio in neurotypical children were also not significant. Like in ASD children, a highly significant negative correlation was obtained between OSI and TAC (*r* = -0.832, *p* = 0.000); however, contrary to those of ASD, a highly positive significant correlation was equally obtained between OSI and TPP in Neurotypical children (*r* = 0.696, *p* = 0.000).

**Table 5:** Correlation of Zn/Cu ratio with Oxidative indices in NT children

Correlations IN NT						
		TPP	TAC	MDA	OSI	Zn/Cu
TPP	Pearson Correlation	1	−.198	−.067	**.696**^**a**^	−.031
	Sig. (2-tailed)		.344	.752	**.000**	.883
	N	25	25	25	25	25
TAC	Pearson Correlation	−.198	1	−.136	**−.832**^**a**^	.216
	Sig. (2-tailed)	.344		.518	**.000**	.300
	N	25	25	25	25	25
MDA	Pearson Correlation	−.067	−.136	1	.040	.241
	Sig. (2-tailed)	.752	.518		.849	.245
	N	25	25	25	25	25
OSI	Pearson Correlation	**.696**^**a**^	**−.832**^**a**^	.040	1	−.190
	Sig. (2-tailed)	**.000**	**.000**	.849		.364
	N	25	25	25	25	25
Zn/Cu	Pearson Correlation	−.031	.216	.241	−.190	1
	Sig. (2-tailed)	.883	.300	.245	.364	
	N	25	25	25	25	25

^a^Correlation is significant at the 0.01 level (2-tailed)

## Discussion

This study was designed to determine the interplay of oxidative stress markers (TAC, TPP, MDA, and OSI) and essential and toxic metals (Mg, Zn, Cu, and Pb) in the pathophysiology of ASD. In this environment, this study was the first to examine oxidative stress markers in children with ASD in Nigeria.

The metabolic function of Mg and Ca has been copiously reported [3, 11, 12]; they are also cofactors in many enzymes in the body including those involved in neurogenesis [13].

In heavy metal toxicity, both Mg and Ca may be displaced by heavy metals like Pb thus changing the molecular configuration of the catalyzing enzyme with its attendant negative consequences. Hence, a reduction in level of these essential metals as seen in this study especially in the central nervous system may initiate a deficit in the neurological functions of the metals. Their deficiency may also elicit replacement in some of the enzymatic processes where they are involved or may exacerbate accumulation of other toxic metals leading to deleterious effect of the latter especially oxidation in sensitive organs like the brain. The observed reduced Mg level in children with ASD in this study may clearly corroborate this. Mg deficiency has been reported to increase NO accumulation and lipid peroxidation and lowers plasma antioxidants levels [6, 14]. Its deficiency has also been reported to increase oxidative stress which was reversed by its supplementation in laboratory animals [15]. Hence, findings in this study were similar to earlier works reporting reduced Mg level in children with ASD as a possible basis of observed lipid peroxidation and increased NO accumulation in this disorder.

Zn is also an essential trace metals involved in catalytic activities of over 300 enzymes and plays a crucial role in protein synthesis, immune system and cell division [16, 17]. Zn deficiency has been reported to predispose individuals to development of neuropsychological changes like emotional imbalance, depression and irritability and cognitive disorders [16]. It has also been reported that reduced Zn level exacerbates Cu toxicity [18]. Because of the very crucial roles of this essential metal in many metabolic activities in the body, downregulation of its level as observed in children with ASD in this study in comparison to what obtained in NT children may be significant in the observed dysfunction and clinical symptoms associated with ASD. The expected role of Zn in protein synthesis and cell division may be easily compromised in these children leading to abnormality in DNA synthesis and its attendant effect on genetic composition especially in the brain. One of the metabolic functions of Zn is its modulatory role on Cu absorption at the intestinal level. The redox potential of Cu is also said to be closely linked with its rate of absorption in the intestine [19]. Aside from its role in redox reactions, Cu is also an important cofactor in many metalloenzymes. These antioxidative functions of Cu as a redox metal are largely dependent on the Zn/Cu combination in metallothionein. This molecule has been implicated as regulator molecules in gene expression, homeostatic control of cellular metabolism of metals, and detoxifying agent against toxic metals.

As observed in this study, there was a downregulation of Zn with the attendant imbalance in the Zn/Cu ratio. This might have precipitated a concurrent reduction in the metallothionein level and a deficit in the function of the latter. The consequence of this may be generation of oxidative stress which the imbalance in Zn/Cu ratio may have induced. The imbalance in the level of the two essential metals has been attributed to pathological changes in the intestinal mucosa in ASD leading to a downregulation of Zn and its consequent impairment of antioxidant defenses and impairment of DNA repair especially in sensitive organs like the brain [16, 20–22]. It has also been previously reported that Zinc induces the intestinal synthesis of a copper-binding protein such as metallothionein. Metallothionein traps copper within intestinal cells and prevents its systemic absorption [23]. Thus, a reduction in the expected Zn level as seen from results of this work may have reduced the synthesis of metallothionein and its implication on the modulatory functions of this Zn/Cu molecule in attenuating toxicity of heavy metals.

In this study, Zn/Cu ratio in NT children was 1:1; although some workers have postulated an inverse relationship in levels of the two metals in NT children [24]; hence, however, the observed reduction in Zn level in children with ASD in this study resulting in a reduction in the ratio may precipitate a reduction in the concentration of metallothionein required for the necessary antioxidative activities of the essential metals. Although results from previous study by Lakshmi and Geetha [25] who found increased Cu levels in ASD, the contradiction may be due to the presence of intestinal malnutrition and malabsorption of Cu in children with ASD in this study. As it has been previously reported, Zn has been associated with Cu absorption in the intestine; unfortunately, presence of malnutrition and/or malabsorption was not investigated in this study.

Hence, it has been proposed that the plasma Zn/serum Cu ratio may be used as a rapid method of determining the functional state of the metallothionein system [26] and by inference the capability of the system to neutralize the toxic effects of heavy metals.

Lead is an established toxicant affecting functions of both central and peripheral nervous systems [27, 28]. Its various domestic and industrial application in the environment and ability to cross biological barrier to accumulate in the internal organs has largely facilitated its toxicity in the body affecting behavior and cognitive functions. The observed elevated Pb level in this study may have been facilitated by the reduction in Zn with the consequent reduction in metallothionein which physiologically could have prevented the accumulation and toxicity of Pb. Results from this study were similar to those of previous studies that reported increased Pb level in children with ASD [29]. The issue of reduced ability to detoxify and eliminate toxic metals like Pb as reported by other workers [19, 30–32] which is largely a function of the amount of metallothionein formed has also been seen in this study.

It may therefore be stated that children with ASD in this study have suffered the pathological consequences of increased oxidative stress due to a reduction in Zn level and the consequent reduction in metallothionein formation leading to accumulation and toxicity of Pb.

Markers of oxidative stress determined in this study were TAC, TPP, MDA, and OSI. The presence of essential metals like Zn, Cu, and Mg in collaboration with other systemic antioxidants was expected to moderate a balance in levels of oxidants and antioxidants to allow for a conducive milieu for normal metabolic activities. This is especially required in sensitive organs like the brain. A reduction in Zn and Mg levels with a concurrent increase in Pb seen in cases in this study may be a good and veritable basis for accumulation of oxidants overwhelming the other antioxidant pathways with the possible creation of an imbalance in the oxidant/antioxidant pool of the body. The reduced TAC level seen in children with ASD in this study could be traced to increased MDA and reduced TPP levels which collectively would overwhelm the antioxidant pool of the body. TPP is an indication of the peroxidase capability in the membrane which with reference to the neuron will have a deleterious effect on its transmission capability. This effect may be accentuated by an increase in MDA level exacerbating the ROS and by implication the oxidation processes at the neural level. The increased Pb level in cases seen in this study may be a clear indication of the source of the increased MDA and reduced TPP which may explain the abnormal cognitive and speech functions characteristic of ASD. Previous studies have reported alteration in composition of fatty acids, oxidation of lipids and phospholipids of the membrane of cells in children with ASD [32, 33]; all these may have direct effect on the functions of proteins that are involved in signal transduction, an important process required in neural transmission.

Elevation of MDA level is suggestive of damage to the lipid component of the cell which may lead to alteration in membrane lipid metabolism, such as composition of fatty acid content of the cell. The ultimate effect of this is its adverse effect on transduction and transmission processes involving these proteins.

Hence, an imbalance in oxidant/antioxidant system of the body especially in sensitive organs like the brain may lead to structural damage due to the deleterious effects of ROS and the attendant disruption in transduction and transmission of signals across neurons. This finding is similar to previous ones where reduced activity of antioxidant enzymes like glutathione peroxidase and superoxide dismutase as well as plasma glutathione concentration was reported in ASD [34–37].

## Conclusion

This study showed that decreased levels of Zn and Mg is a possible etiological basis for accumulation of Pb leading to increase in oxidative stress with a reduction in TAC and the attendant abnormal neurological sequelae associated with children with ASD in this environment.

## Limitations

Due to the level of understanding of research benefits in the African setting, it has not been easy recruiting participants for this study; this has thus caused the limited sample size of the study. Lack of funding for the project ostensibly due to the same reason has also affected the scope of works carried out in this project. Also, for cultural reasons, non-invasive methods like nail or hair analysis could not be used in this environment. The use of blood analysis in this project has greatly affected the sample size and by implication some of the statistical tools that could be applied in the analysis of results. The sociodemographic data obtained from participants in the study might have indicated variations in the socioeconomic status of the babies while corrections could not be made for multiple comparisons arising from the above differences.

## Data Availability

All data in support of this manuscript are attached and available.
